# Lifestyle intervention program in deprived obese adult patients and their non-deprived counterparts

**DOI:** 10.1371/journal.pone.0188297

**Published:** 2017-11-16

**Authors:** Celine Loddo, Emilie Pupier, Rémy Amour, Maud Monsaingeon- Henry, Kamel Mohammedi, Blandine Gatta-Cherifi

**Affiliations:** 1 Department of Endocrinology, Diabetology and Nutrition, University Hospital of Bordeaux, Haut Leveque Hospital, Pessac, France; 2 University of Bordeaux, U.F.R. of medical sciences, Bordeaux, France; 3 Institut National de la Santé et de la Recherche Médicale (INSERM), Neurocentre, France Magendie, Physiopathologie de la Plasticité Neuronale, Bordeaux, France; "INSERM", FRANCE

## Abstract

**Introduction:**

Although it is known that the prevalence of obesity is high in deprived patients, the link between deprivation and obesity, and the impact of deprivation on compliance and efficacy of a lifestyle intervention program are not known.

**Materials and methods:**

Deprivation was assessed in 40 patients (23 Females, mean±SD age: 49±17 years) from the diabetology department and 140 patients (101 Females, age: 50±15 years) from the nutrition department of Bordeaux University hospital. Eighty-seven patients suffering from obesity were evaluated before and after a tailored, multidisciplinary lifestyle intervention. Deprivation was assessed using EPICES scores. Deprivation was defined with an EPICES score > 30.

**Results:**

Deprived patients suffering from obesity had significantly higher current (43.8 ±8.4 versus 40.9 ± 5.5 kg/m^2^, p = 0,02) and maximal BMI (46.1± 8.6 versus 42.3± 5.2 kg/m^2^, p = 0.002) compared to non-deprived obese. Percentage of body weight loss was not different according to deprivation (4.74 ± 0.75 versus 4.65 ± 1.04%, p = 0.9). EPICES scores were not different according to adherence to lifestyle intervention program (20.5 ± 8.5 versus 29.9 ± 3.9 versus 29.0 ±2.5, no follow up versus partial follow up versus total follow up, p = 0,58).

**Conclusion:**

Deprived patients suffering from obesity have a more serious disease than non-deprived patients. However, neither compliance to the lifestyle intervention program nor body weight loss differed between deprived patients with obesity and non-deprived ones. Deprivation should not be a limitation when enrolling patients with obesity in lifestyle intervention programs.

## Introduction

The prevalence of obesity keeps increasing in industrialized countries [1]. The prevalence of obesity increased drastically last decades across the world including Europe and it incidence has been expected to reach a very high rate by 2030[2]. Indeed, the prevalence of obesity has dramatically increased in people over 65 years but on the other hand, obesity is also concerning children and teenagers [3]. In addition, obesity is linked to worldwide socioeconomic inequalities with a higher prevalence of obesity in populations with low educational levels or low income [3–5].

The concept of deprivation is defined by P. Townsend as a ‘state of observable and demonstrable disadvantage relative to the local community or the wider society to which an individual, family or group belongs’. The prevalence of deprivation is also increasing and is the main cause of health inequalities [6].

Deprivation has been linked to poor metabolic control in patients with diabetes.The link between deprivation and obesity is known [4,7,8] as well as its impact on the efficiency of lifestyle intervention programs. Therefore, the main goals of our study was to characterize deprivation in subjects suffering from obesity, and to analyze if adhesion to lifestyle intervention programs and the expected weight loss differ according to the presence of deprivation measured with EPICES score. Indeed, all patients suffering from obesity were enrolled in a lifestyle intervention program, which allowed us to verify whether deprived obese gave up the program more frequently or lost less weight.

We also had the opportunity to estimate deprivation with EPICES score in a small group of patients who had been referred to Diabetes department in the same hospital.

## Material and methods

This study was designed and conducted in the department of Endocrinology, Diabetology and Nutrition of Bordeaux University Hospital, France. Part of the department is devoted to Endocrinology (pituitary, adrenal and thyroid diseases, neuroendocrine tumors), another part is responsible for the care of type 1 and type 2 diabetes; while the nutrition part is especially involved in the care of patients suffering from obesity.

The French Ministry of Health labeled this nutrition department as CSO (Centre Spécialisé pour la prise en charge de l’Obésité) in 2012. A multidisciplinary team composed of endocrinologists, dieticians, psychologists, physical activity coaches and nurses is specifically involved in the care of patients with severe obesity. These patients are referred to CSO by various health care professionals, primarily general practitioners but also by cardiologists, rheumatologists, and others medical doctors.

During the first consultation with the patient at the outpatient clinic, the program and assessment are explained by the endocrinologist and the following care is then decided together. Patients with BMI > 40 kg/m2 or > 35 kg/m2 with comorbidities are proposed to be included in an “educational week” as in-patients. During this week, inquiry of physical symptoms, physical examination, fasting blood examination, and blood pressure (BP) monitoring are conducted. Eating habits, physical activity, psychological wellbeing and sleep habits are assessed by the dietician, the physical activity coaches/physiotherapist, the psychologist and the endocrinologist respectively. All the professionals involved in the program have been especially trained in the field of obesity and therapeutic patient education. During the week, patients have to participate in both individual and group sessions conducted by the different professionals: nurses, dieticians, physicals activity coaches/ physiotherapist, psychologists and endocrinologist. This later conducts a group session to inform the patients of the medical aspects of obesity and its complications. The interdisciplinary team discusses information obtained from the assessment during the week and a care plan is proposed for each patient, taking into account the specific needs of each individual. By focusing on small step-by-step lifestyle improvements, the program aims to convert the lifestyle changes to daily habits in order that they become routine. Deprivation was measured during this week.

Modalities of follow-up after this week were as following. All patients are proposed to join a multidisciplinary follow up out patients program for a theoretical total duration of one year that consist of weekly sessions for a total duration of ten weeks. Each week, sub-groups of 10 patients met the endocrinologist, dieticians, physicals activity coaches and nurses on specifics workshops. After this 10 weeks, a follow up on day hospital at 3, 6 and 9 months (who the patient meets the medical staff) is proposed except for those whose bariatric surgery occurred after the weekly sessions. A nurse manages convocations by mails and phone calls (up to 2 times if patient doesn’t respond).

### Participant characteristics

All patients entering the educational week between November 2013 and June 2015 described above were considered for inclusion in this study except for those who could not fill in the questionnaires due to the language barrier. We also randomly included 40 patients from the diabetology department at the same time.

Some patients from the obesity unit were suffering from diabetes while patients suffering diabetes who were in-patients in the diabetology department could also suffer from obesity. Patients issued from the part of the department devoted to the care of diabetes are named as patients from the diabetology department while patients coming from the department devoted to the care of obesity will be named as patients suffering from obesity all along the manuscript.

Anthropometric data were collected while patients were barefoot and wearing only underwear. Weight was determined using the same digital scale during the entire program. Height was measured using a measuring rod. At each session, the same care assistant measured the anthropometric data for all patients of each sub-group. Body mass index (BMI) was calculated. Waist circumference was measured with a non-elastic tape-lint at the end of a natural breath at midpoint between the top of the iliac crest and the lower margin of the last palpable rib by the same care assistant all patient of each sub-group.

Fasting serum total cholesterol, HDL-cholesterol, LDL-cholesterol, triglycerides, blood glucose, were determined with the analyzer AU 2700 BCO Bexman Coulter Olympus.

Education level was defined according to 4 categories: no diploma, college degree, high school diploma or more. Physical activity was scored out of 20. It included a fitness score out of 10, methods of locomotion scored out of 5 and sport activity scored out of 5 [9,10] as well as assessment of balance, of muscle strength and of suppleness. The objective of the physical activity score is to determine the current status and to evaluate the improvement during the follow-up. According to the score, a specific care by a physiologist can be proposed. Information about when obesity started (in childhood, adolescence or adulthood) was recorded with declarative data or growth curves from the health journal if available.

For patients suffering from obesity, others data were collected. Indeed, number of approaches was defined by the previously number of weight loss attempts (alone or with the help of dieticians or doctors) according to declarative data. Consumption of alcohol and tobacco were also determined. All patients entering the program signed an informed consent.

### Deprivation

Deprivation was assessed using the Evaluation de la Precarite et des Inegalites de sante dans les Centres d’Examens de Sante [Evaluation of Precarity and Inequalities in Health Examination Centers (EPICES)] score computed on the basis of individual conditions of deprivation [11]. The EPICES score was developed in 2002 and was based on a first questionnaire of 42 items selected by a panel of French experts from National Health Insurance relative to dimensions of deprivation as defined by Wrezinski and Townsend [6,12]. A factorial correspondence analysis identified 11 salient items on which calculation of the EPICES score is based: marital status (one item), health insurance status (one item), economic status (three items), family support (three items) and leisure activity. The score is computed by adding each question co- efficient to intercept whenever the answer is ‘yes’. The higher the score, the more deprived the patient is. EPICES score was validated in a large cohort of 197 389 people [13]. Different thresholds have been used to define deprivation since the creation of the EPICES score. Therefore thresholds from 30,17 [14] to 38,5 [15] or even 40 [16] have been used. In this study the cutoff of 30, chosen by the Centre Technique d’appui et de Formation (CETAF) of health centers and the health insurance, was used to define deprivation. In the present study the EPICES questionnaire was administered during a short interview during hospitalization for all participants.

### Statistical analyses

Statistical analyses were performed using Prism 6 (GraphPad Sofware, Inc). The EPICES score was used as a dichotomous variable to divide participants into two subgroups: non-deprived (≤30) and deprived (>30). Comparisons of patient characteristics by deprivation status were performed using χ^2^ tests or ANOVA as appropriate. Association between BMI and deprivation status was also evaluated using linear regression analysis after adjustment for sex and age. Correlations of biological, and metabolic variables with EPICES score were calculated by Pearson coefficients. Results are expressed as mean ± SD or numbers and percent. A P-value < 0.05 indicated statistical significance.

## Results

Forty (23 females, 17 males, mean±SD age: 49 ± 17 years) patients from the diabetology department and 140 (101 females, 39 males; age: 50 ± 15 yrs) patients suffering from obesity were included. EPICES scores (mean±SD) were 29.8 ± 23.0 and 29.42 ±19.41 in patients from the diabetology department and obese respectively.

### Patients from the diabetology department

Briefly, patients from the diabetology department had significantly higher fasting plasma glucose compared to those from nutrition department (1.31 ± 0.65 versus 1.06 ± 0.32, g/l, patients, p<0.05) and HbA1c [8.0% ± 1.5 versus 6.0% ± 1.0, patients from the diabetology department versus patients suffering from obesity, p<0.05]. Patients from the diabetology department had significantly lower BMI compared to patients suffering from obesity (42.3 ± 7.2 versus 29.6 ±7.2, kg/m^2^, p<0.05). Among patients from the diabetology department, we did not observe any significant difference between deprived and non-deprived patients for HbA1C, hypertension, dyslipidemia or tobacco use.

### Patients suffering from obesity

One hundred and forty patients suffering from obesity (101 females, 39 males) aged 49 ± 13 years were included. Mean±SD weight, BMI, and waist circumference were 115.9 ± 25.7 kg; 42.3 ± 7.2 kg/m^2^; and 124 ± 18 cm, respectively. Twenty two percent had diabetes, 44% and 47% were using lipids lowering and antihypertensive drugs, respectively. Obesity had started during childhood for 16% of patients, during adolescence for 14% and during adulthood for 70%.

The main±SD EPICES score of patients suffering from obesity was 29.42 ± 19.41. Sixty-seven patients (47.8%) had an EPICES score > 30 and were classified as deprived (Table 1). Among patients suffering from obesity, deprived patients had both higher current (43.8 ±8.4 versus 40.9 ± 5.5 kg/m^2^, p = 0,02) and maximal BMI (46.1± 8.6 versus 42.3± 5.2 kg/m^2^, p = 0.002) compared to non-deprived patients. These differences remain significant after adjustment for age and sex. There was a positive correlation between EPICES score and current BMI (r = 0,2056, p = 0.01), maximal BMI (r = 0.2592, p = 0.002) and maximal weight (r = 0.1842, p = 0.03). Deprived patients suffering from obesity tended to have a larger waist circumference (127 ± 21 versus 121 ±13 cm, p = 0.05). The prevalence of hypertension, dyslipidemia, increased liver enzymes and type 2 diabetes was not significantly different between deprived and non-deprived obese (Table 1). There was a negative correlation between EPICES score and HDL cholesterol (r = -0.2029, p = 0.02).

**Table 1:** Characteristics of non-deprived patients suffering from obesity (N = 73) versus deprived ones (N = 67).

	Non-deprived Patients (EPICES < 30)	Deprived Patients (EPICES > 30)	p
**Number of patients**	73	67	
**Women (%)**	71	71	NS
**Age (ans)**	50 ± 13	48 ± 12	NS
**BMI (kg/m^2^)**	40,9 ± 5,5	43,8 ± 8,4	**0,02**
**Weight (kg)**	112,2 ± 19,1	120,0 ± 30,1	0,07
**Maximum BMI (kg/m^2^)**	42,3 ± 5,2	46,1 ± 8,6	**0,002**
**Maximum Weight (kg)**	116,4 ± 19,4	126,0 ± 30,1	**0,03**
**Waist circumference (cm)**	121 ± 13	127 ± 21	**0,05**
**Number of approaches:**			
**1** **2–4** **5 and +**	33 16 24	26 17 24	NS
**Onset obesity:**			
**Childhood** **Teenage** **Adulthood**	8 12 53	15 9 43	NS
**Education level:**			
**No diplôma** **College degree** **High school diploma** **More**	4 25 15 17	7 28 10 8	NS
**T2DM**	16	15	NS
**Fasting glucose (g/l)**	1,04 ± 0,24	1,09 ± 0,39	NS
**HbA1C (%)**	5,89 ± 0,80	6,1 ± 1,3	NS
**Patients on antihypertensive drugs (%)**	31	35	NS
**Patients on hypolipemic drugs (%)**	30	32	NS
**Triglycerides (g/l)**	1,38 ± 0,60	1,48 ± 0,71	NS
**HDL (g/l)**	0,50 ± 0,12	0,48 ± 0,15	NS
**LDL (g/l)**	1,30 ± 0,35	1,26 ± 0,39	NS
**AST (UI/l)**	33 ± 16	32 ± 18	NS
**ALT (UI/l)**	39 ± 21	40 ± 23	NS
**GGT (UI/l)**	57 ± 56	58 ± 59	NS
**Current smoking**	10	13	NS
**Alcohol consumption**	3	4	NS
**Patients on treatment for sleep apnea (%)**	22	20	NS
**Physical activity score**	8 ± 4	7 ± 4	NS

Results are expressed as mean ± SD; NS: non significant; T2DM: type 2 Diabetes mellitus; BMI: body mass index; *AST*: *aspartate aminotransferase; ALT*: *alanine aminotransferase; GGT*: *gamma glutamyl transferase; HDL*: *High density lipoprotein; LDL*: *Low density lipoprotein*

Deprivation did not differ according to sex. Deprived obese women had significantly higher maximal BMI compared to non-deprived (45,3 ± 6.9 versus 42.0 ± 5.4 kg/m^2^, p = 0.01) while this was only a trend in males (48.2 ± 12.0 versus 43.2 ± 4.2 kg/m2, p = 0.07). In addition, deprived men patients suffering from obesity had a higher waist circumference compare to non-deprived (147 ± 24 versus 132 ±9, p = 0.048).

### Follow-up of patients suffering obesity

**Fig 1:**
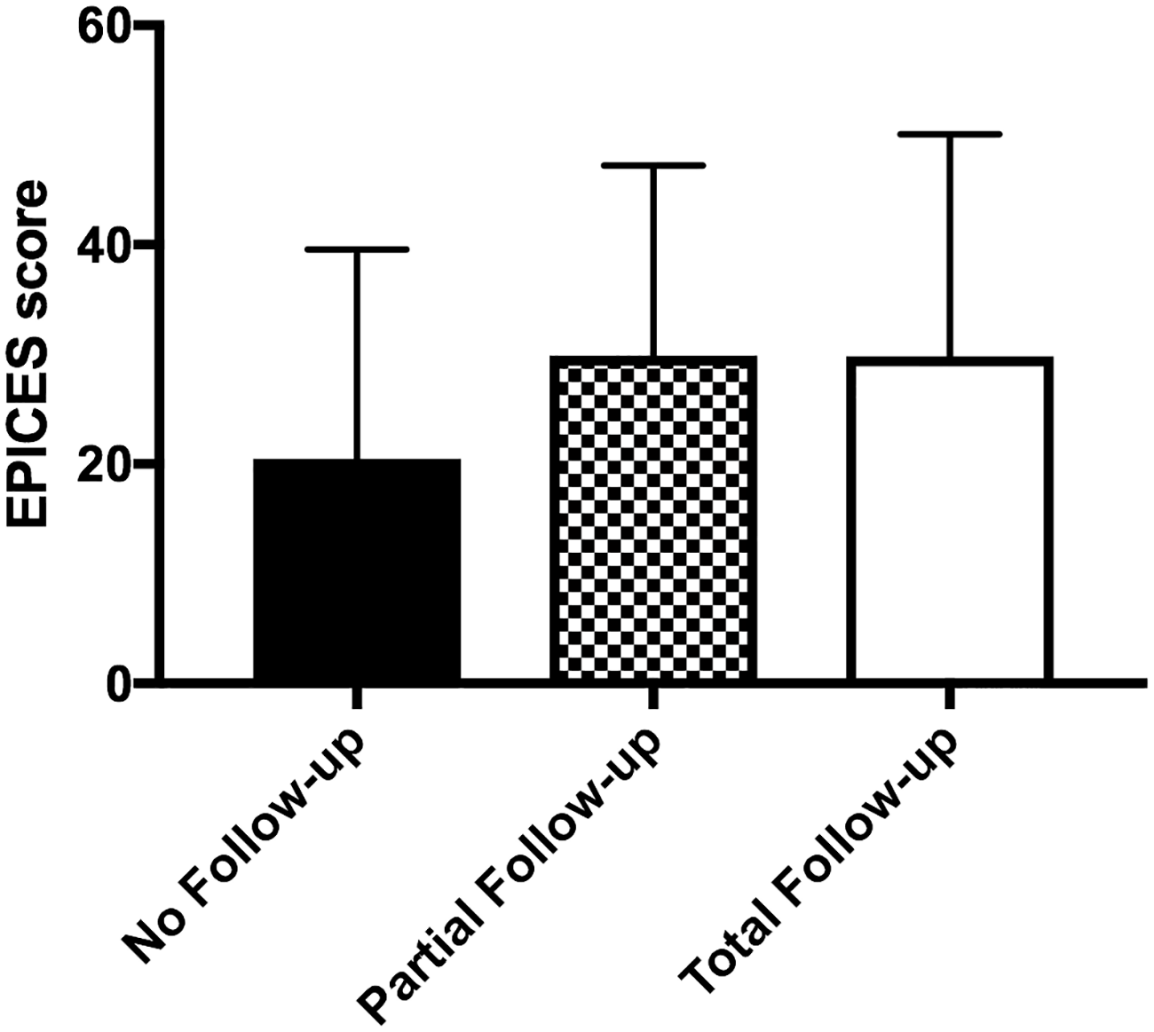
EPICES score according to duration of follow up.

Follow-up was available for the first 87 enrolled participants suffering from obesity: 5 (5.7%) patients never returned after the educational week, 20 (23%) patients came back but gave up follow-up before the end (i.e patient came at least once but didn’t follow the entire planned follow-up) while 62 (71.3%) patients suffering from obesity completed the entire duration of the prescribed one year follow-up. Obesity had started earlier in patients who gave up the program before the end compared to those who did not. EPICES score was not different between patients who followed the entire program compared to those who followed it only partly, and those who never came to the follow-up (29.02 ±2.5 versus 29.88 ± 3.88 versus 20.48 ± 8.5, total versus partial versus no follow-up, p = 0.58) (Fig 1). For patients who completed the whole follow-up, HbA1C (percentage of improvement of HbA1C: 9.6 ± 16.6% versus 1.7 ± 5.9%, p = 0.03) and triglycerides (percentage of decreased of triglycerides: 19.0 ±19.2% versus 6.1 ± 24.5%, p = 0.049) were significantly more improved in deprived patients than in non-deprived ones. In the group who gave up follow-up before the end, physical activity score was less improved in deprived than in non-deprived patients (-1 ± 3 versus +3 ± 4 points, p = 0.03).

**Fig 2:**
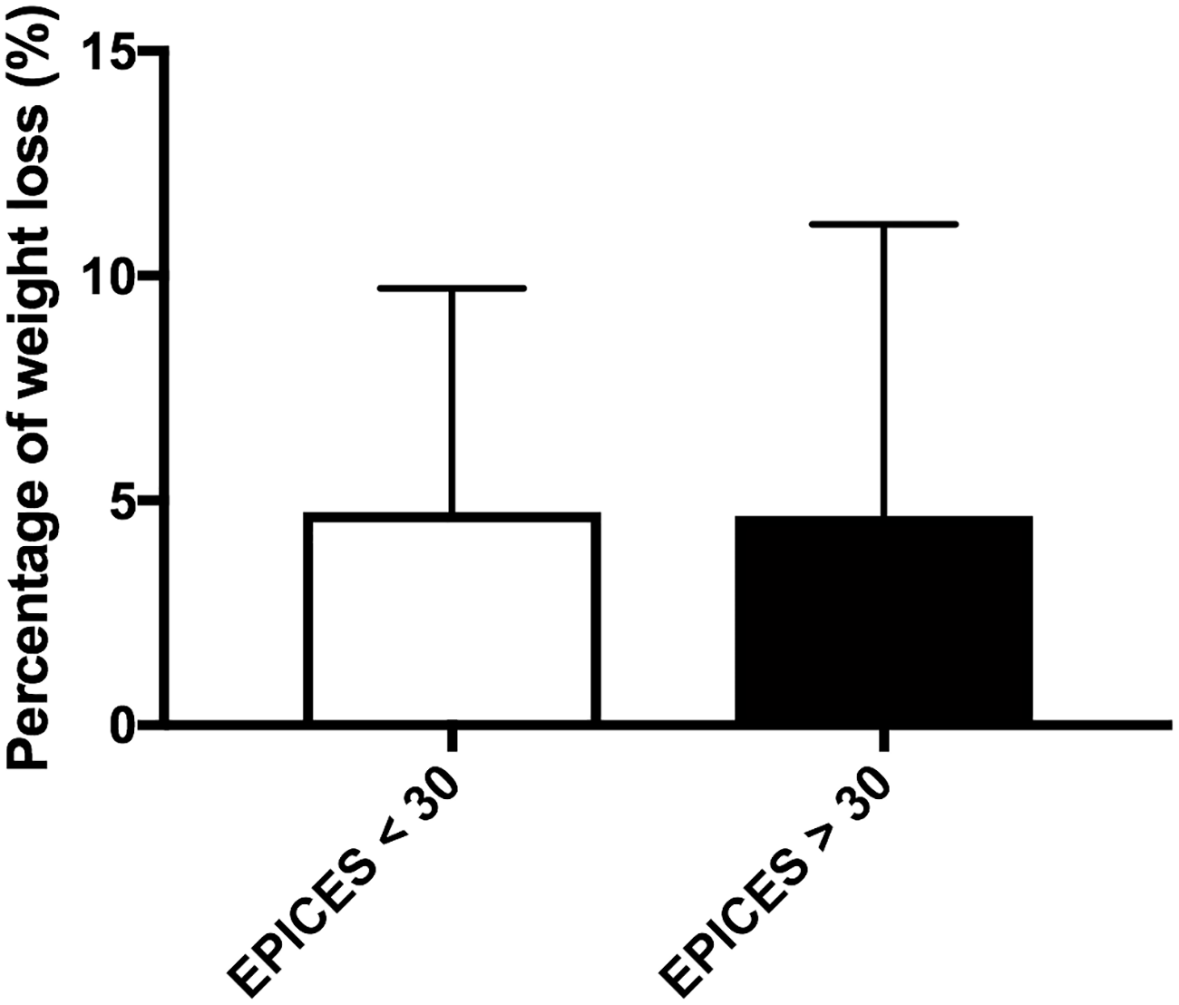
Percentage of weight loss according to EPICES score.

The mean weight loss for all patients engaged in the program who entered the follow-up program was 4.8 ± 5.7% of body weight. Waist circumference significantly decreased between the beginning and the end of the program (124 ± 19 cm versus 118 ± 13 cm, p = 0.01) while there was a trend toward a BMI decrease (43.0 ± 8.2 versus 40.8 ± 8.3 kg/m2, p = 0.09). Body weight loss was not different in deprived patients compared to non-deprived ones (4.74 ± 0.75 versus 4.65 ± 1.04, p = 0.9) (Fig 2).

## Discussion

### Deprivation and obesity

This is the first study that measures the impact of deprivation, estimated by the validated EPICES score, on concordance with a care program for patients with obesity. We did not find any difference of EPICES score according to follow up—i.e. EPICES score was not different between patients that never returned compared to those who gave up before the end or to those who completed the entire follow-up. The overall compliance is therefore quite good and does not differ according to deprivation. Differently, Moulin et al. previously published that deprived patients were significantly more prone to give up medical and dental follow-up [17]. This poor compliance has also been described for gynecologic care [13]. In the field of metabolic disease, it also seems that patients suffering from diabetes with a better socio-economic status do have better access to care [18]. Brown et al. suggested many underlying mechanisms that can explain poor compliance in deprived situations. The health inequalities observed for people with diabetes at different levels of the socioeconomic hierarchy may be explained by differences in one or more individual-level characteristics, including patient-provider communication, culture and acculturation, mental health, social support, and stress [18]. Our results do not confirm this poor compliance among deprived patients. Obesity is often relegated to the bottom of the problem list of the disease [19]. We can suggest that patients suffering from obesity are not often proposed to join this kind of multidisciplinary programs especially if they are deprived. Therefore, when deprived patients suffering from obesity are proposed to enter these kinds of lifestyle intervention programs, they are maybe more enthusiastic and have better compliance than expected.

In addition, we have also shown that although deprived patients had a more serious disease, they lost the same amount of body weight as the non-deprived patients. Therefore, we measured for the first time the impact of deprivation on body weight loss and showed that body weight loss was no different between deprived and non-deprived patients. Of note, the amount of body weight lost by our patients has been previously shown to be significantly associated with the improvement of obesity linked-comorbidities [20]. Therefore, our results suggest that including deprived patients suffering from obesity in lifestyle intervention programs is as efficient in terms of body weight loss as it is for non-deprived counterparts. Deprivation does not have to be an obstacle when enrolling deprived patients with obesity in these kinds of programs. This a useful and optimistic message when the prevalence of obesity keeps increasing especially in industrialized countries and while it is well known that obesity is more prevalent in deprived patients [21,22]. Obesity is one of the main priorities of public health, in an attempt to combat this issue there has been an increase in the setup of many lifestyle intervention programs and our results suggest that these programs would benefit deprived patients that are often more concerned by obesity.

Interestingly, we have shown that deprived patients suffering from obesity who gave up the program before the end improved their physical activity score less than the non-deprived ones. This is coherent with the idea that geographical location can play a role in the inequalities regarding access to care. Deprived people usually live in areas where the environment is less favourable for health. Of note, environments with more cycle paths increase activity levels and decrease the use of motor vehicles [23], which in turn is good for health. We can assume that deprived patients with obesity were living in environments that did not facilitate the access to physical activity. Those who completed the whole follow-up increased their physical activity thanks to the hospital program while those who gave up the program most likely did not partake in physical activity in their neighborhood. Therefore, in addition to lifestyle intervention programs that will help fight against obesity, public health politics that allow easy access to physical activity in all areas like sidewalks everywhere are really mandatory.

The underlying mechanisms of this link between obesity and deprivation are complex. They include health behaviors, neighbourhood, health status and psychological factors [24]. Bad eating habits are very likely to be involved. Indeed, food intake is typically low in protein, fruits, and vegetables but high in lipids. Unhealthy processed foods are less expensive and more accessible for deprived patients. The term « food insecurity » is therefore proposed when access to healthy food is limited due to financial problems [25–29].

### Deprivation and diabetes

The link between deprivation and diabetes has been described previously [22,30]. Bihan et al also used the same tool to characterize deprivation in patients with diabetes [14,15]. In both Bihan’s studies, the EPICES score of patients suffering from diabetes was 33.2 ± 20.7 and 38.9 ± 22.9 respectively [14,15]. In our cohort, the EPICES score of patients with diabetes was 29.42 ± 19.41. However, our cohort was composed by patients suffering from both type 1 and type 2 diabetes while Bihan et al. only studied patients with type 2 diabetes [14,15]. Although both Bihan’s studies and ours were performed in France, the formers were conducted in the department of Seine Saint Denis when deprivation is more prevalent than here in Gironde [14,15,31–33]. Of note, Bihan et al previously found that deprived patients with diabetes had higher BMI than non-deprived ones, suggesting that patients suffering from obesity could have a higher EPICES score [14].

### Limitations of the study

Some limitations of the study have to been highlighted and make our conclusions interpreted with cautions. The most important limitation of our work is the small sample size of the study population, especially for the follow-up data. Therefore, the lack of differences of body-weight loss and duration of follow-up has to be really interpreted with caution. It would be interesting to try to replicate our findings in a larger group of patients. In addition, due to the small number of patients, our study may thus suffer from a lack of power to detect some modest differences and may contain some type 2 errors. For instance, the lack of higher prevalence of metabolic complications of obesity in deprived patients with obesity does not preclude an absence of a real difference. One other limit of our study is that we only included in-patients that were in a university hospital and these patients may be more severe and more deprived than the general population. Moreover, these patients were enrolled in a specific multidisciplinary care program labelled by the minister of Health and our results may not be replicated for other programs. We can however suggest that these types of programs should be developed. Lastly, we have only studied the influence of deprivation on patients included in a medical program for obesity and did not analyze if deprivation changes access to bariatric surgery in these patients. Therefore, socioeconomic deprivation has been recently shown to be a significant barrier in the choice of bariatric surgery [34].

Furthermore, we used EPICES score for measuring deprivation as a dichotomous variable divided into two groups according to the value 30. This is a way to use EPICES score [14,35]. However, population studied seem to be globally a deprived population and perhaps the use of EPICES score as quartiles or a continuous variable would have highlighted different results on the impact of deprivation, both on health status, compliance, or result of intervention. Although, EPICES score has many advantages, it would have been also interesting to use an ecological indicator like European Deprivation Index, or Townsend and Carstairs indexes [6,36,37], especially to explore the link with physical activity.

## Conclusion

In conclusion, we show that deprived patients suffering from obesity have a more serious disease than non-deprived ones. However, deprived patients with obesity benefit equally from lifestyle intervention programs, as do their non-deprived counterparts.
